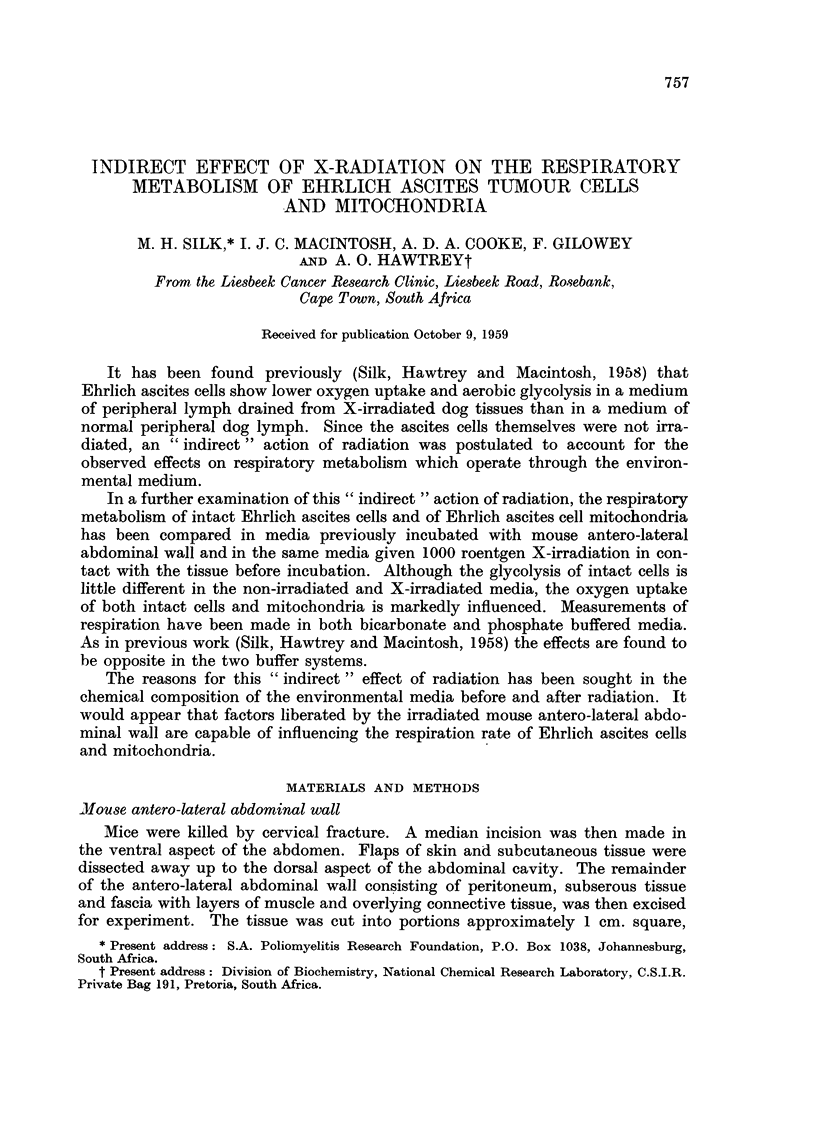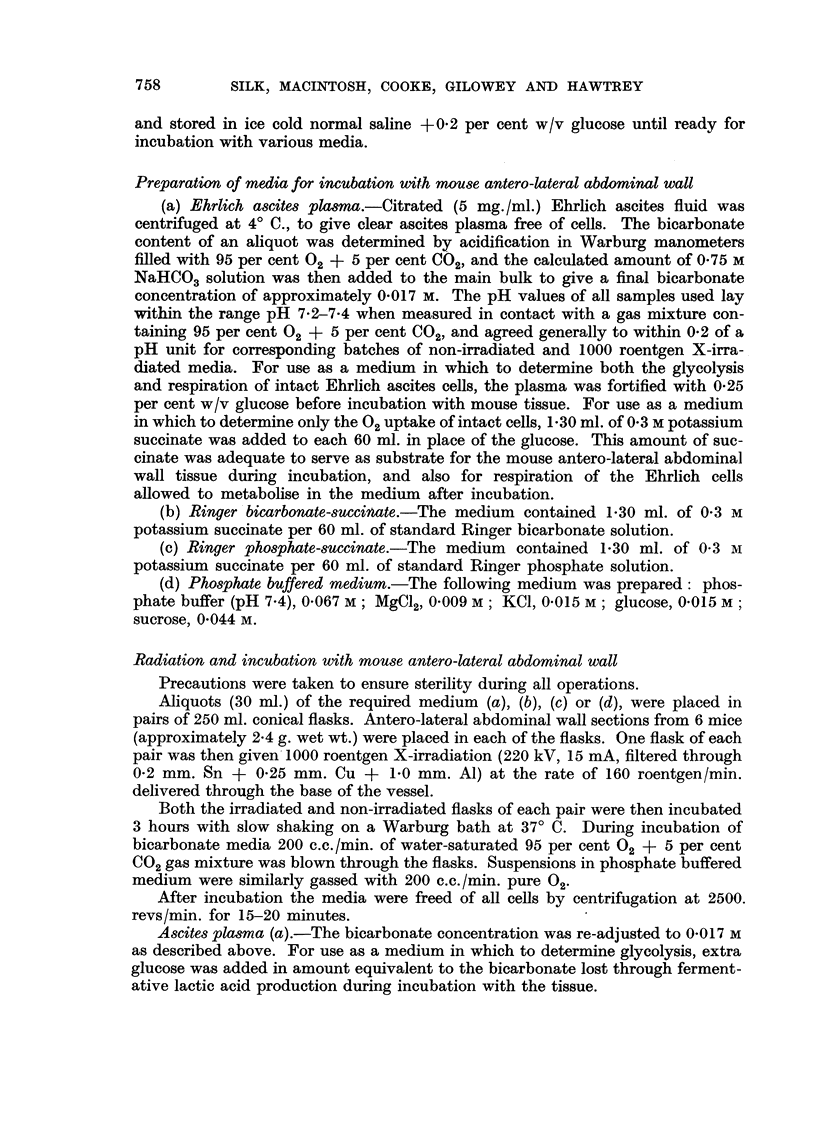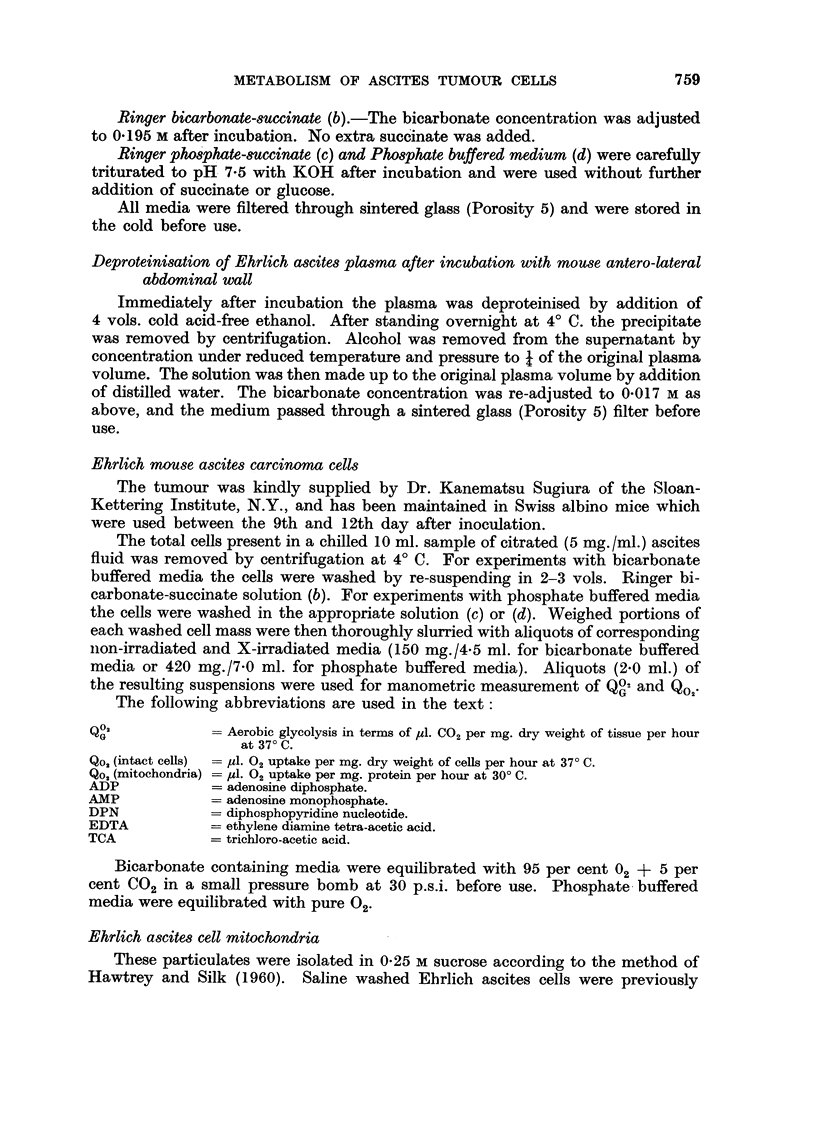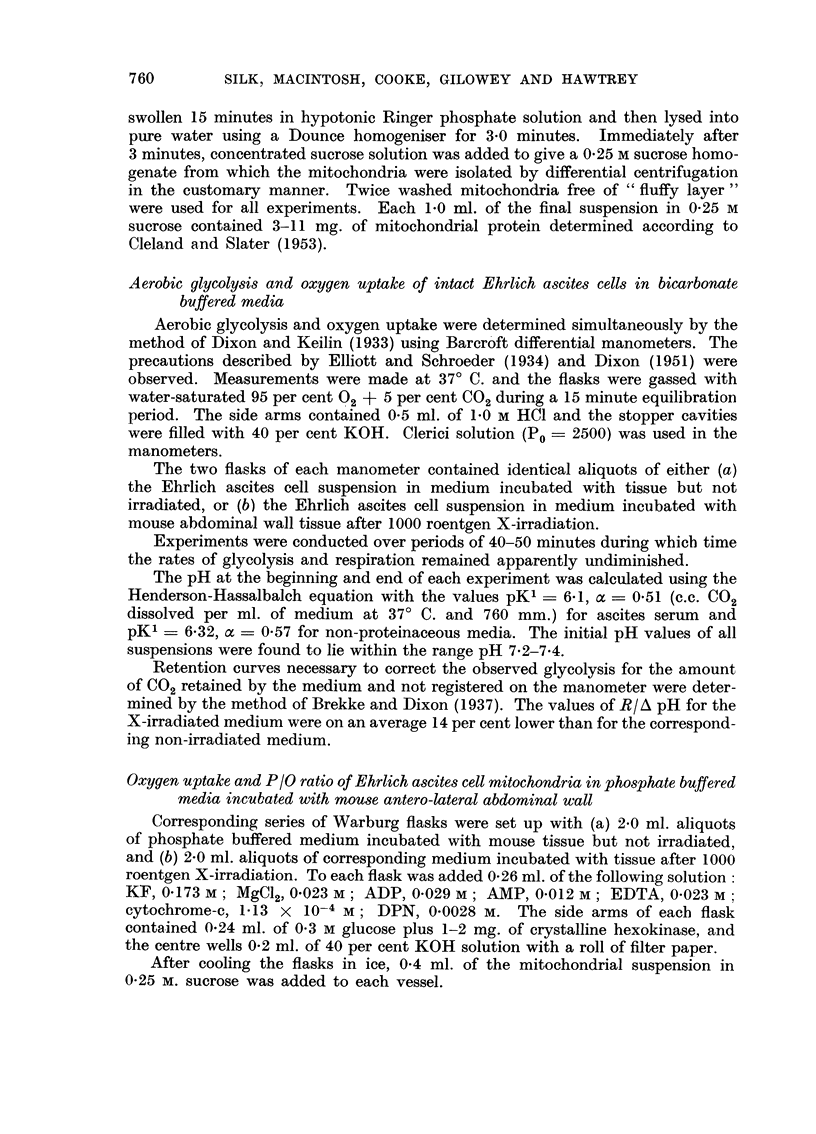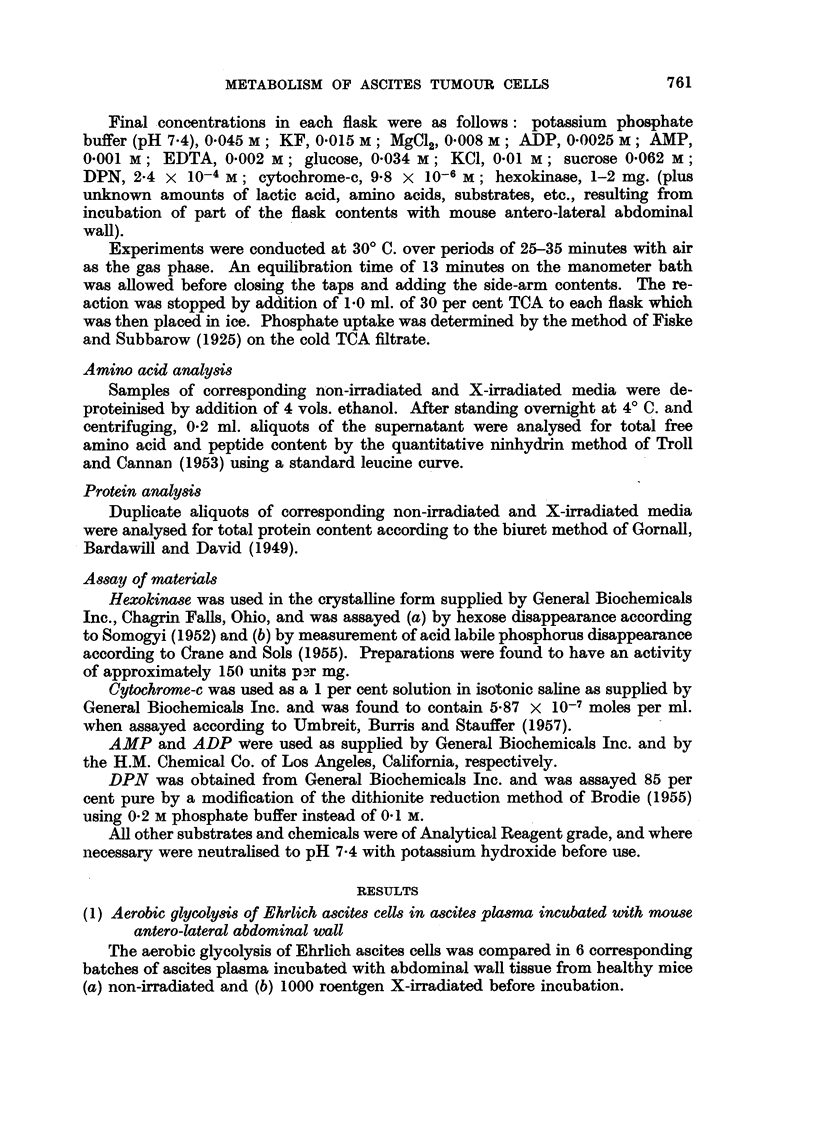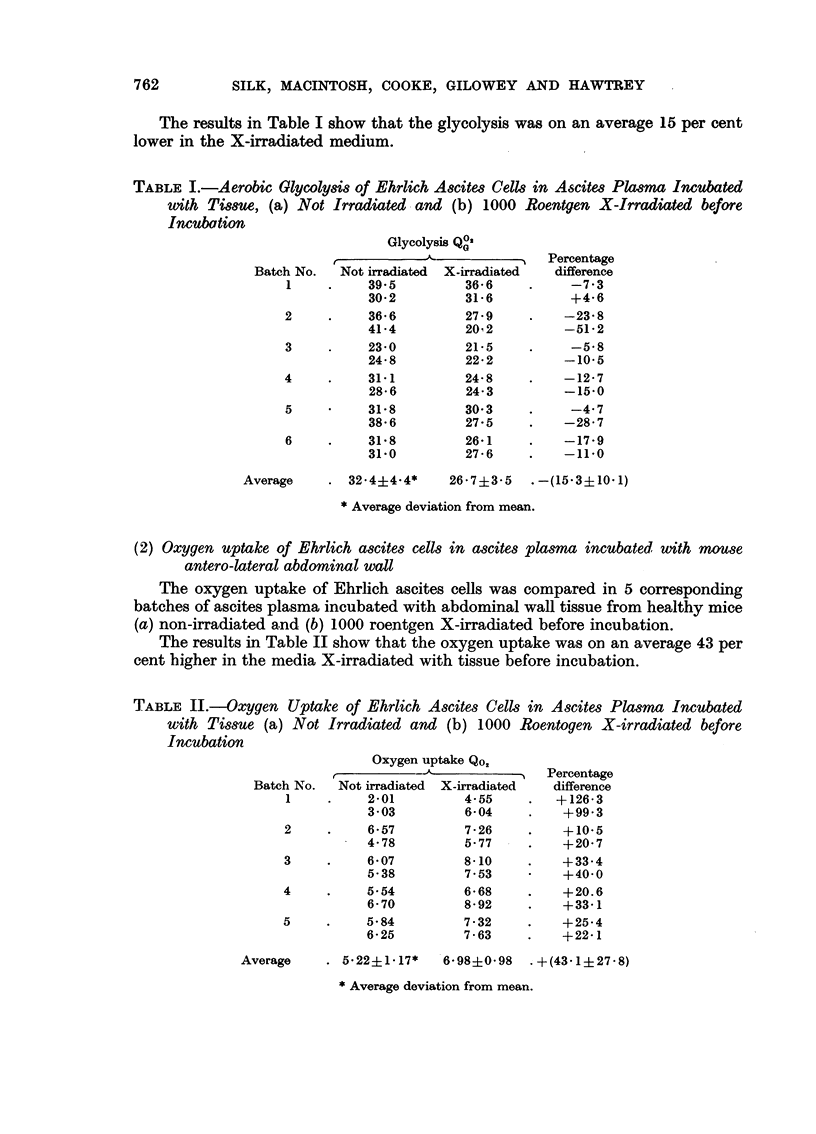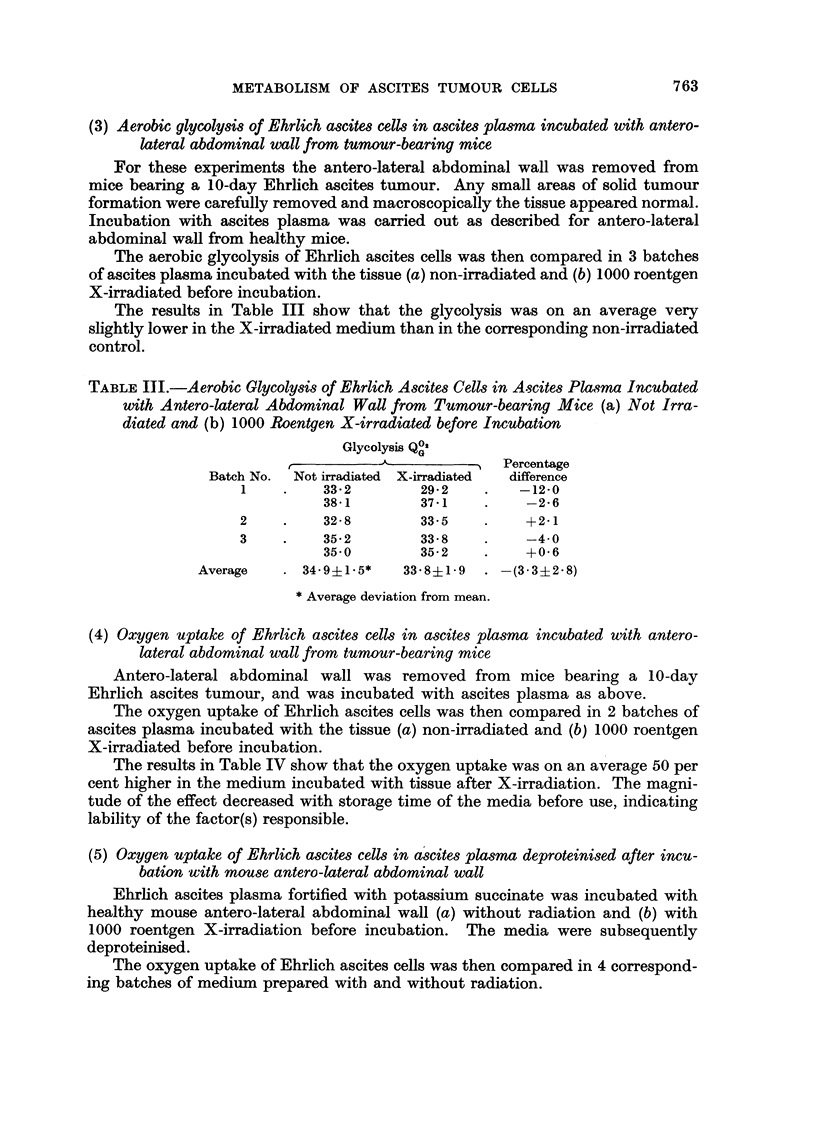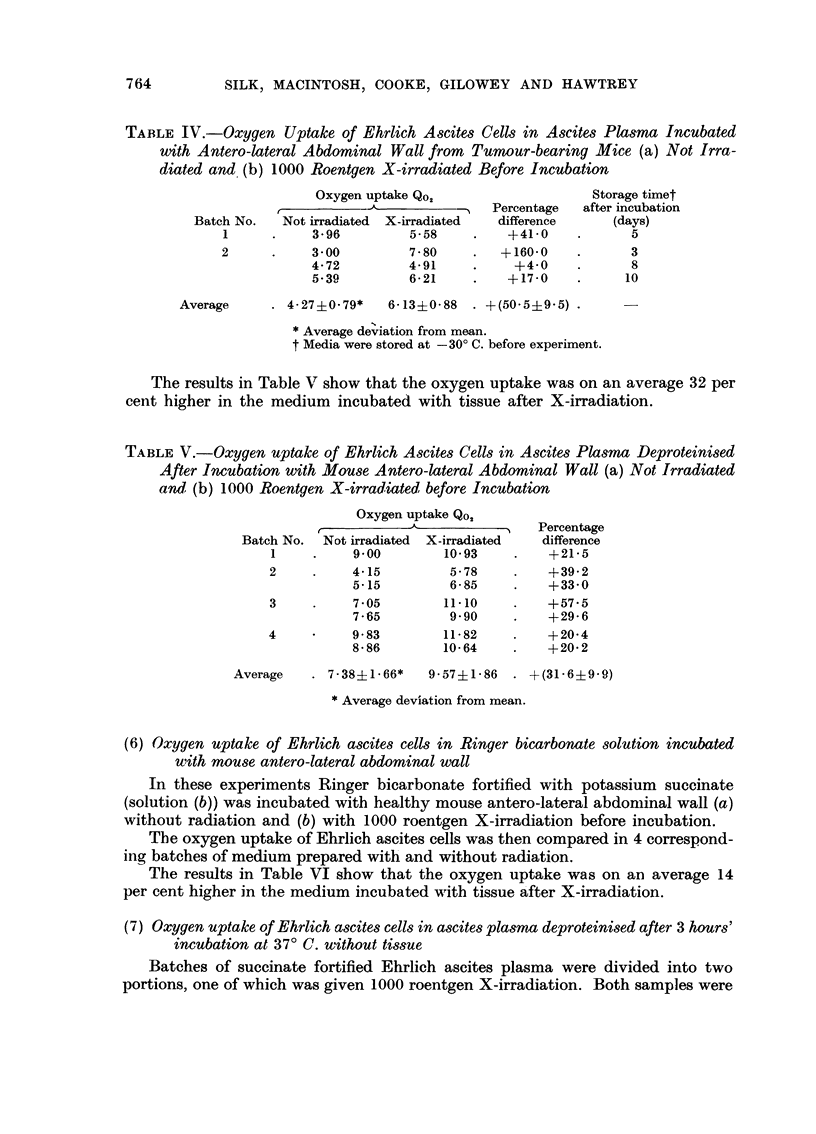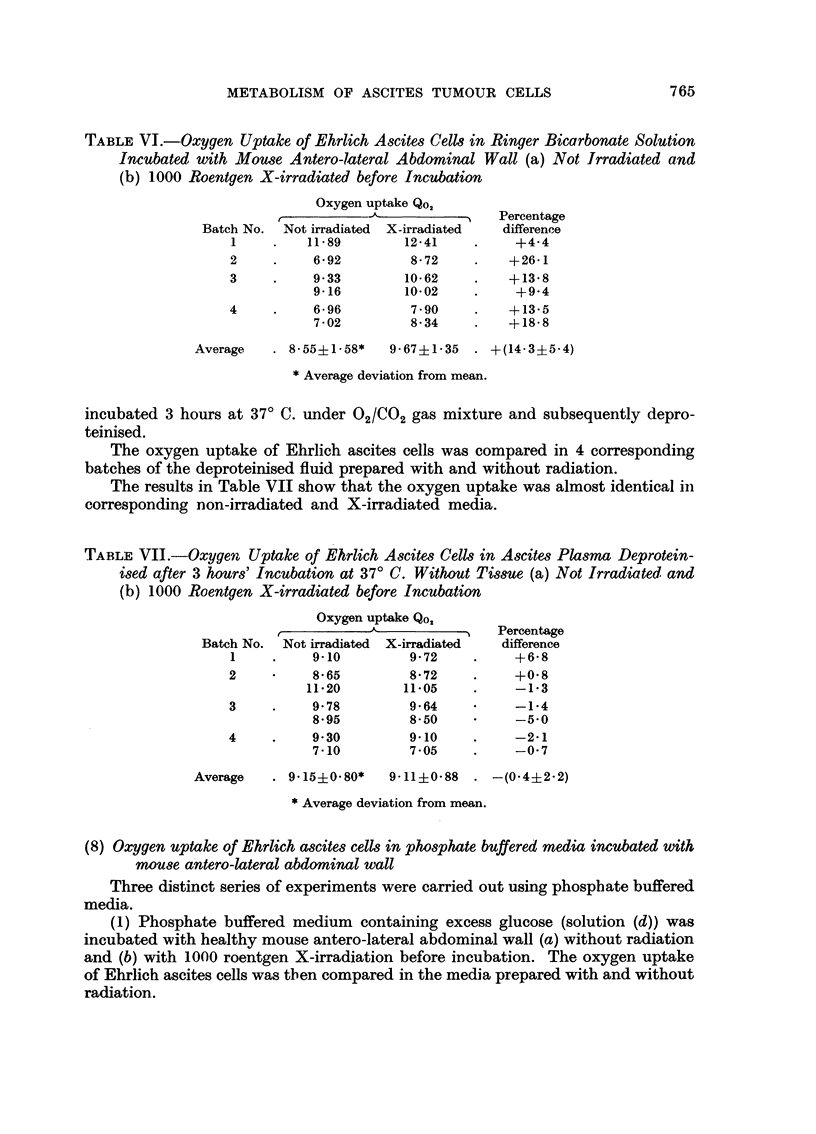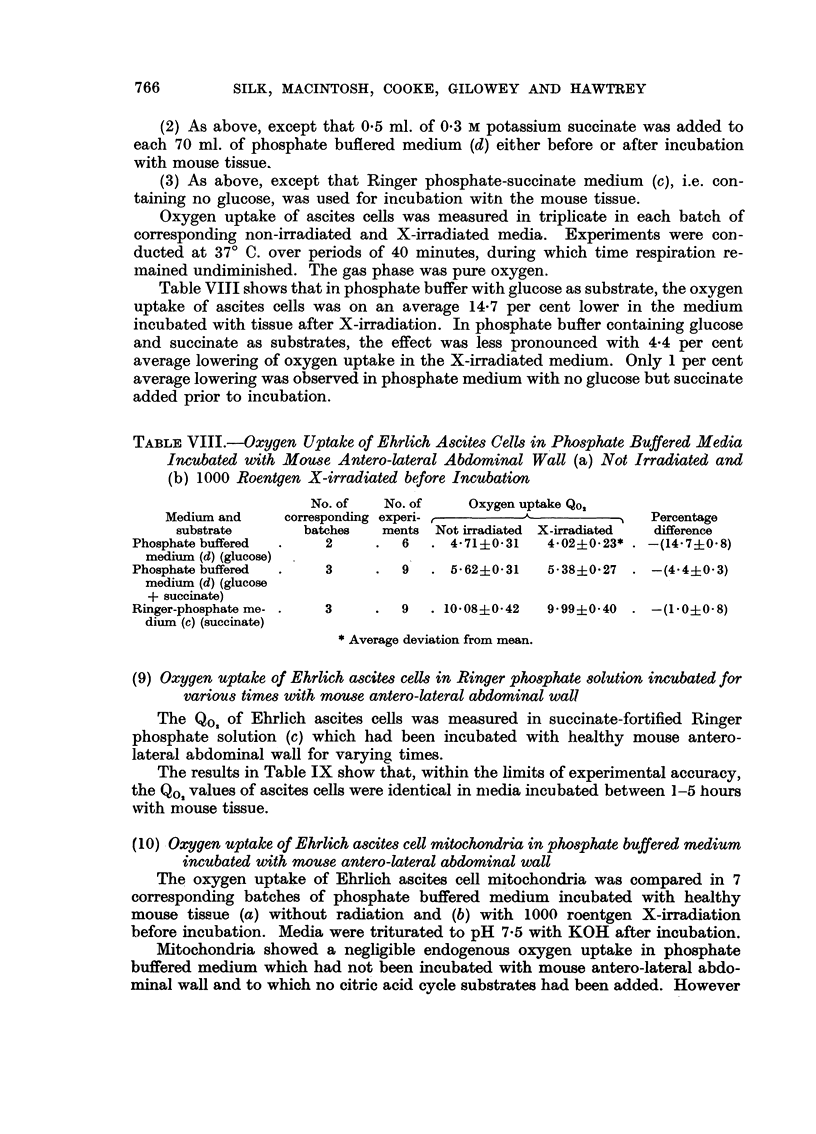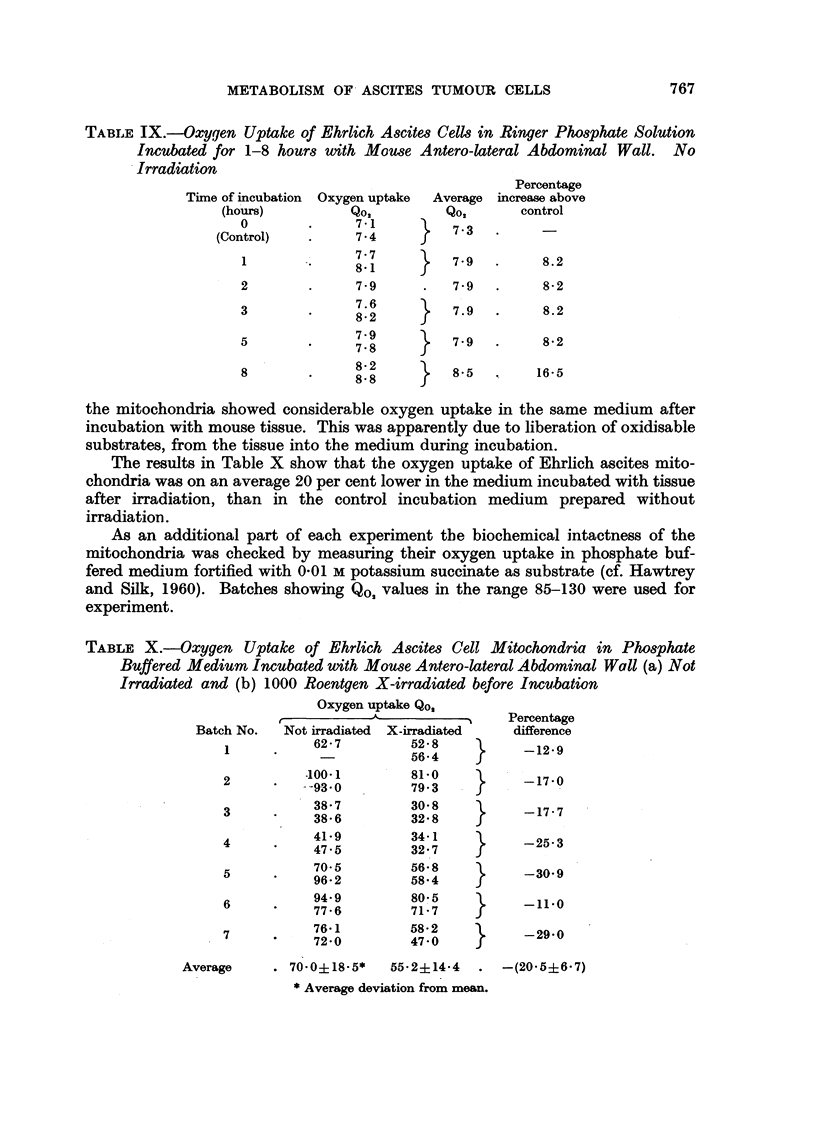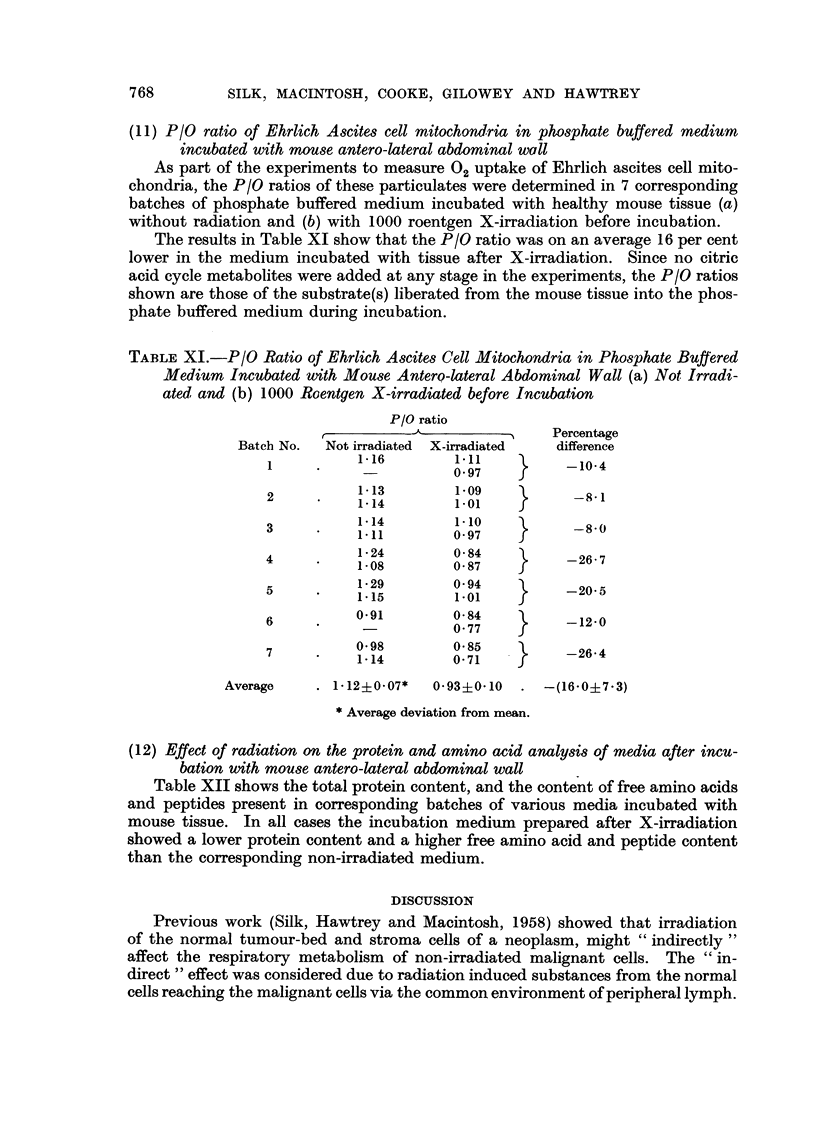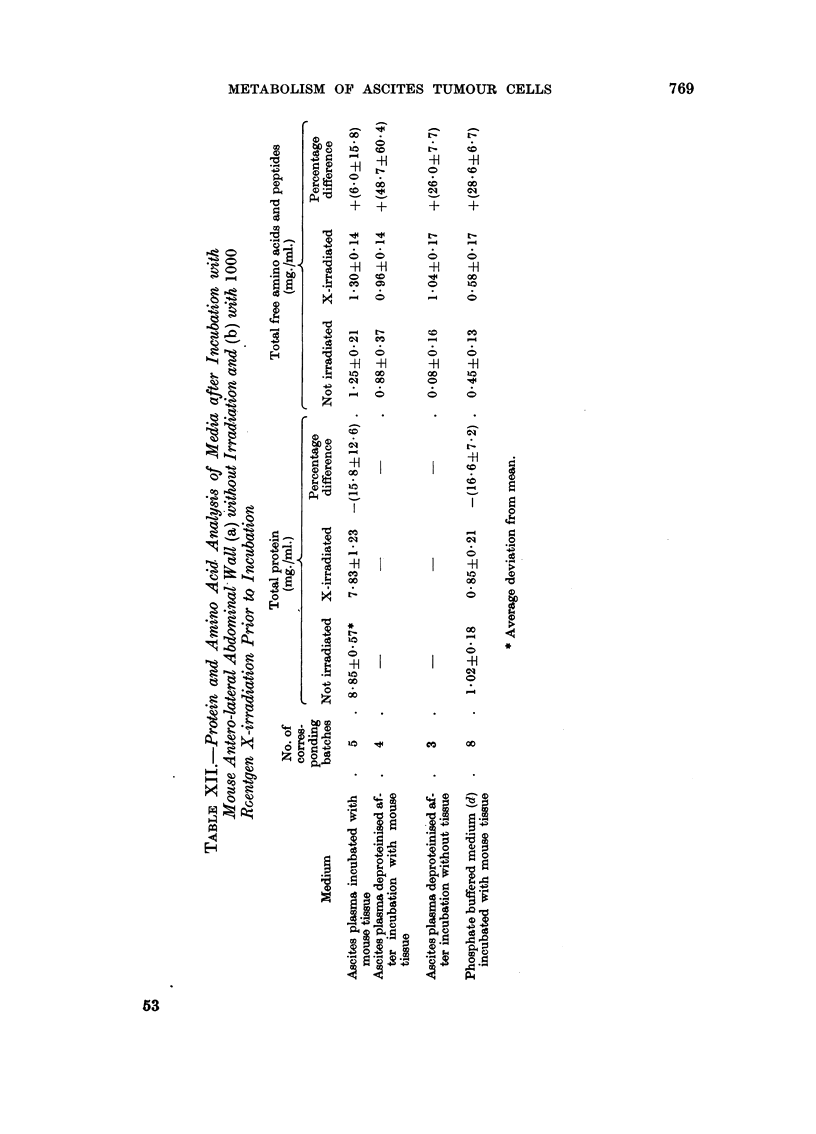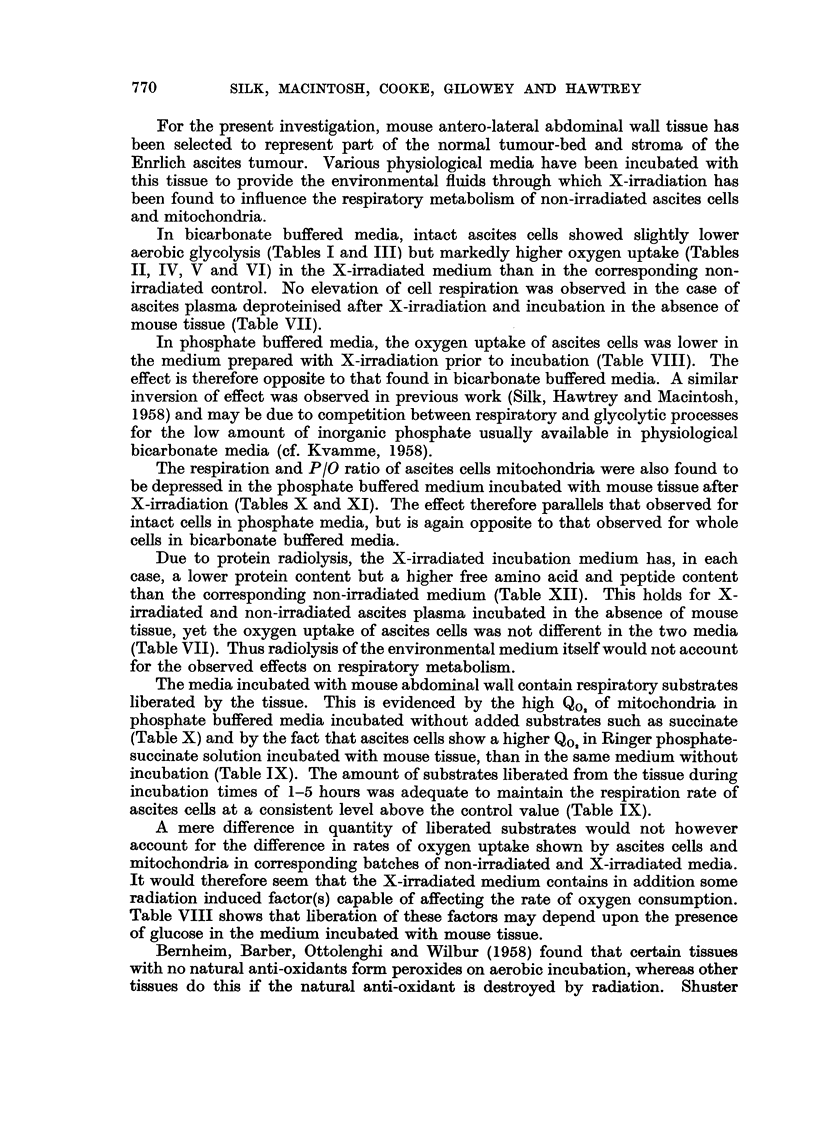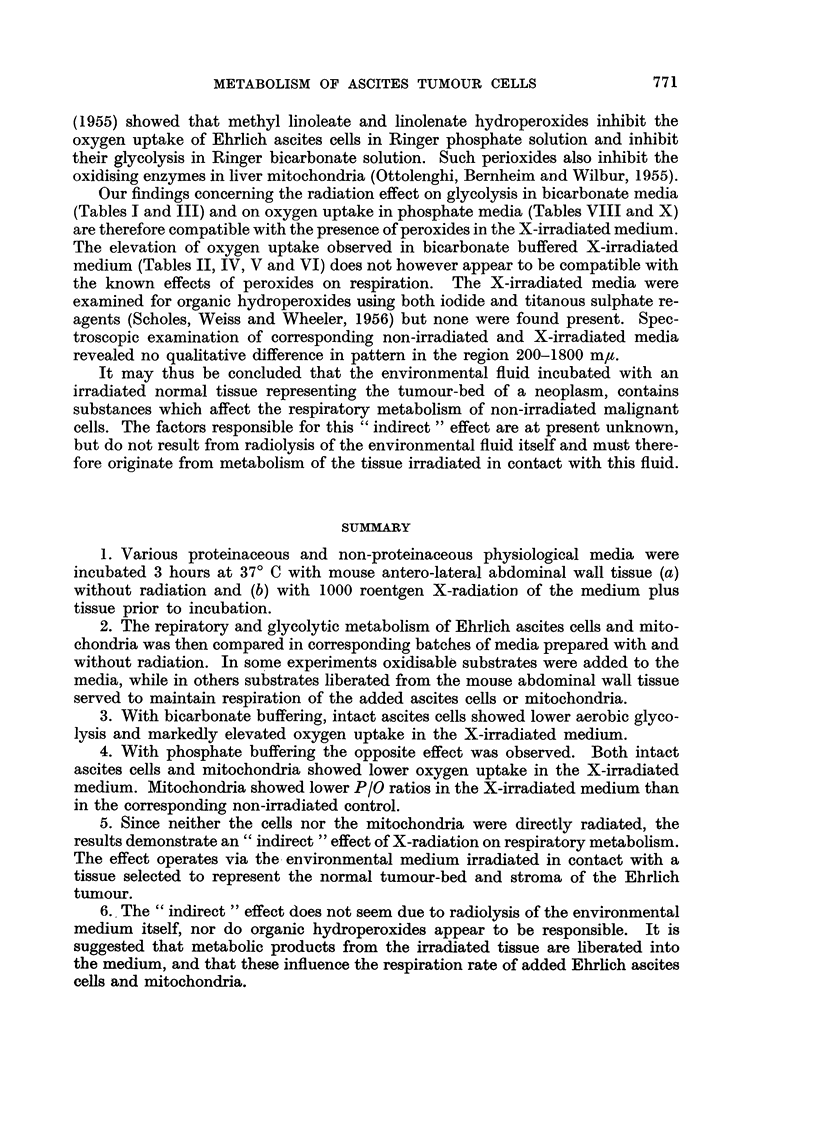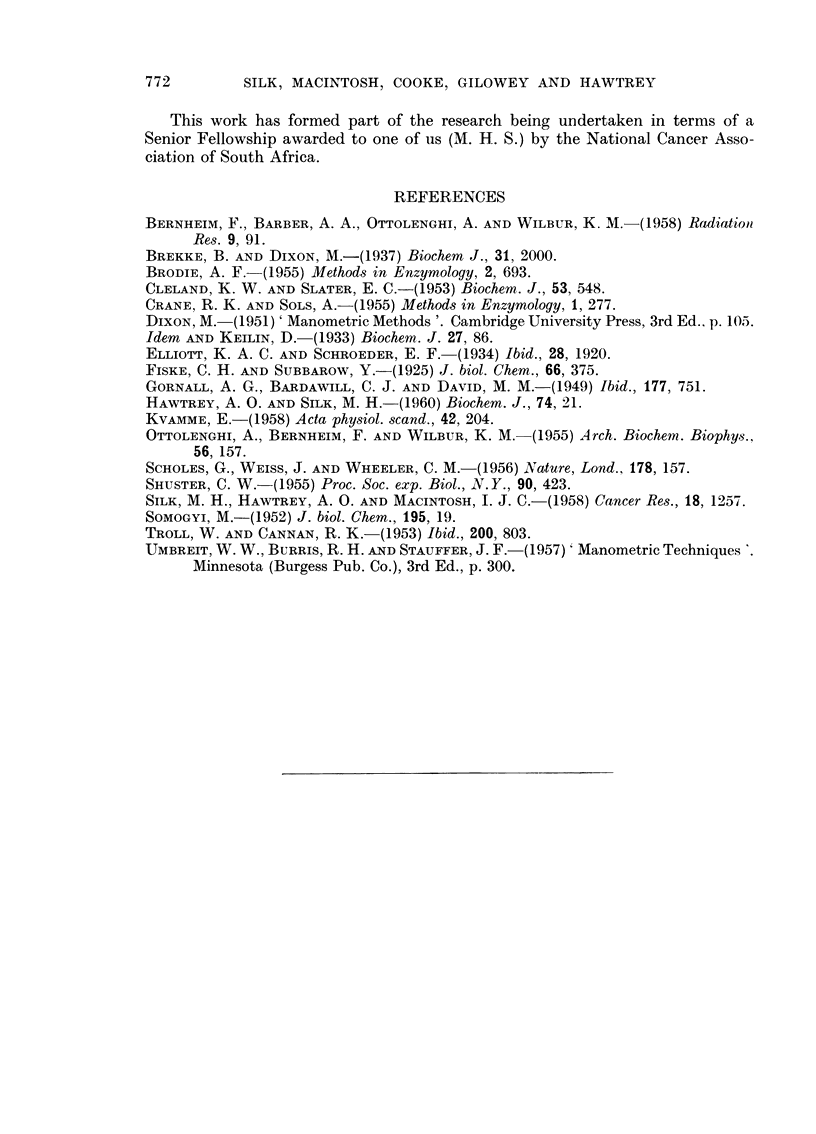# Indirect Effect of X-Radiation on the Respiratory Metabolism of Ehrlich Ascites Tumour Cells and Mitochondria

**DOI:** 10.1038/bjc.1959.85

**Published:** 1959-12

**Authors:** M. H. Silk, I. J. C. Macintosh, A. D. A. Cooke, F. Gilowey, A. O. Hawtrey


### INDIRECT EFFECT OF X-RADIATION ON THE RESPIRATORY METABOLISM OF EHRLICH ASCITES TUMOUR CELLS AND MITOCHONDRIA

M. H. SILK,* I. J. C. MACINTOSH, A. D. A. COOKE, F. GILOWEY AND A. O. HAWTREY†

*From the Liesbeek Cancer Research Clinic, Liesbeek Road, Rosebank, Cape Town, South Africa*

Received for publication October 9, 1959

It has been found previously (Silk, Hawtrey and Macintosh, 1958) that Ehrlich ascites cells show lower oxygen uptake and aerobic glycolysis in a medium of peripheral lymph drained from X-irradiated dog tissues than in a medium of normal peripheral dog lymph. Since the ascites cells themselves were not irradiated, an " indirect " action of radiation was postulated to account for the observed effects on respiratory metabolism which operate through the environmental medium.

In a further examination of this " indirect " action of radiation, the respiratory metabolism of intact Ehrlich ascites cells and of Ehrlich ascites cell mitochondria has been compared in media previously incubated with mouse antero-lateral abdominal wall and in the same media given 1000 roentgen X-irradiation in contact with the tissue before incubation. Although the glycolysis of intact cells is little different in the non-irradiated and X-irradiated media, the oxygen uptake of both intact cells and mitochondria is markedly influenced. Measurements of respiration have been made in both bicarbonate and phosphate buffered media. As in previous work (Silk, Hawtrey and Macintosh, 1958) the effects are found to be opposite in the two buffer systems.

The reasons for this " indirect " effect of radiation has been sought in the chemical composition of the environmental media before and after radiation. It would appear that factors liberated by the irradiated mouse antero-lateral abdominal wall are capable of influencing the respiration rate of Ehrlich ascites cells and mitochondria.

### MATERIALS AND METHODS

### *Mouse antero-lateral abdominal wall*

Mice were killed by cervical fracture. A median incision was then made in the ventral aspect of the abdomen. Flaps of skin and subcutaneous tissue were dissected away up to the dorsal aspect of the abdominal cavity. The remainder of the antero-lateral abdominal wall consisting of peritoneum, subserous tissue and fascia with layers of muscle and overlying connective tissue, was then excised for experiment. The tissue was cut into portions approximately 1 cm. square,

* Present address: S.A. Poliomyelitis Research Foundation, P.O. Box 1038, Johannesburg, South Africa.

† Present address: Division of Biochemistry, National Chemical Research Laboratory, C.S.I.R. Private Bag 191, Pretoria, South Africa.

and stored in ice cold normal saline +0·2 per cent w/v glucose until ready for incubation with various media.

*Preparation of media for incubation with mouse antero-lateral abdominal wall*

(a) *Ehrlich ascites plasma.*—Citrated (5 mg./ml.) Ehrlich ascites fluid was centrifuged at 4° C., to give clear ascites plasma free of cells. The bicarbonate content of an aliquot was determined by acidification in Warburg manometers filled with 95 per cent $O_2$ + 5 per cent $CO_2$, and the calculated amount of 0·75 M $NaHCO_3$ solution was then added to the main bulk to give a final bicarbonate concentration of approximately 0·017 M. The pH values of all samples used lay within the range pH 7·2–7·4 when measured in contact with a gas mixture containing 95 per cent $O_2$ + 5 per cent $CO_2$, and agreed generally to within 0·2 of a pH unit for corresponding batches of non-irradiated and 1000 roentgen X-irradiated media. For use as a medium in which to determine both the glycolysis and respiration of intact Ehrlich ascites cells, the plasma was fortified with 0·25 per cent w/v glucose before incubation with mouse tissue. For use as a medium in which to determine only the $O_2$ uptake of intact cells, 1·30 ml. of 0·3 M potassium succinate was added to each 60 ml. in place of the glucose. This amount of succinate was adequate to serve as substrate for the mouse antero-lateral abdominal wall tissue during incubation, and also for respiration of the Ehrlich cells allowed to metabolise in the medium after incubation.

(b) *Ringer bicarbonate-succinate.*—The medium contained 1·30 ml. of 0·3 M potassium succinate per 60 ml. of standard Ringer bicarbonate solution.

(c) *Ringer phosphate-succinate.*—The medium contained 1·30 ml. of 0·3 M potassium succinate per 60 ml. of standard Ringer phosphate solution.

(d) *Phosphate buffered medium.*—The following medium was prepared : phosphate buffer (pH 7·4), 0·067 M ; $MgCl_2$, 0·009 M ; KCl, 0·015 M ; glucose, 0·015 M ; sucrose, 0·044 M.

*Radiation and incubation with mouse antero-lateral abdominal wall*

Precautions were taken to ensure sterility during all operations.

Aliquots (30 ml.) of the required medium (*a*), (*b*), (*c*) or (*d*), were placed in pairs of 250 ml. conical flasks. Antero-lateral abdominal wall sections from 6 mice (approximately 2·4 g. wet wt.) were placed in each of the flasks. One flask of each pair was then given 1000 roentgen X-irradiation (220 kV, 15 mA, filtered through 0·2 mm. Sn + 0·25 mm. Cu + 1·0 mm. Al) at the rate of 160 roentgen/min. delivered through the base of the vessel.

Both the irradiated and non-irradiated flasks of each pair were then incubated 3 hours with slow shaking on a Warburg bath at 37° C. During incubation of bicarbonate media 200 c.c./min. of water-saturated 95 per cent $O_2$ + 5 per cent $CO_2$ gas mixture was blown through the flasks. Suspensions in phosphate buffered medium were similarly gassed with 200 c.c./min. pure $O_2$.

After incubation the media were freed of all cells by centrifugation at 2500. revs/min. for 15–20 minutes.

*Ascites plasma* (*a*).—The bicarbonate concentration was re-adjusted to 0·017 M as described above. For use as a medium in which to determine glycolysis, extra glucose was added in amount equivalent to the bicarbonate lost through fermentative lactic acid production during incubation with the tissue.

*Ringer bicarbonate-succinate* (*b*).—The bicarbonate concentration was adjusted to 0·195 M after incubation. No extra succinate was added.

*Ringer phosphate-succinate* (*c*) *and Phosphate buffered medium* (*d*) were carefully triturated to pH 7·5 with KOH after incubation and were used without further addition of succinate or glucose.

All media were filtered through sintered glass (Porosity 5) and were stored in the cold before use.

*Deproteinisation of Ehrlich ascites plasma after incubation with mouse antero-lateral abdominal wall*

Immediately after incubation the plasma was deproteinised by addition of 4 vols. cold acid-free ethanol. After standing overnight at 4° C. the precipitate was removed by centrifugation. Alcohol was removed from the supernatant by concentration under reduced temperature and pressure to ¼ of the original plasma volume. The solution was then made up to the original plasma volume by addition of distilled water. The bicarbonate concentration was re-adjusted to 0·017 M as above, and the medium passed through a sintered glass (Porosity 5) filter before use.

*Ehrlich mouse ascites carcinoma cells*

The tumour was kindly supplied by Dr. Kanematsu Sugiura of the Sloan-Kettering Institute, N.Y., and has been maintained in Swiss albino mice which were used between the 9th and 12th day after inoculation.

The total cells present in a chilled 10 ml. sample of citrated (5 mg./ml.) ascites fluid was removed by centrifugation at 4° C. For experiments with bicarbonate buffered media the cells were washed by re-suspending in 2–3 vols. Ringer bicarbonate-succinate solution (*b*). For experiments with phosphate buffered media the cells were washed in the appropriate solution (*c*) or (*d*). Weighed portions of each washed cell mass were then thoroughly slurried with aliquots of corresponding non-irradiated and X-irradiated media (150 mg./4·5 ml. for bicarbonate buffered media or 420 mg./7·0 ml. for phosphate buffered media). Aliquots (2·0 ml.) of the resulting suspensions were used for manometric measurement of $Q_G^{O_2}$ and $Q_{O_2}$.

The following abbreviations are used in the text :

| | |
|---|---|
| $Q_G^{O_2}$ | = Aerobic glycolysis in terms of $\mu$l. $CO_2$ per mg. dry weight of tissue per hour at 37° C. |
| $Q_{O_2}$ (intact cells) | = $\mu$l. $O_2$ uptake per mg. dry weight of cells per hour at 37° C. |
| $Q_{O_2}$ (mitochondria) | = $\mu$l. $O_2$ uptake per mg. protein per hour at 30° C. |
| ADP | = adenosine diphosphate. |
| AMP | = adenosine monophosphate. |
| DPN | = diphosphopyridine nucleotide. |
| EDTA | = ethylene diamine tetra-acetic acid. |
| TCA | = trichloro-acetic acid. |

Bicarbonate containing media were equilibrated with 95 per cent $0_2$ + 5 per cent $CO_2$ in a small pressure bomb at 30 p.s.i. before use. Phosphate buffered media were equilibrated with pure $O_2$.

*Ehrlich ascites cell mitochondria*

These particulates were isolated in 0·25 M sucrose according to the method of Hawtrey and Silk (1960). Saline washed Ehrlich ascites cells were previously swollen 15 minutes in hypotonic Ringer phosphate solution and then lysed into pure water using a Dounce homogeniser for 3·0 minutes. Immediately after 3 minutes, concentrated sucrose solution was added to give a 0·25 M sucrose homogenate from which the mitochondria were isolated by differential centrifugation in the customary manner. Twice washed mitochondria free of " fluffy layer " were used for all experiments. Each 1·0 ml. of the final suspension in 0·25 M sucrose contained 3–11 mg. of mitochondrial protein determined according to Cleland and Slater (1953).

*Aerobic glycolysis and oxygen uptake of intact Ehrlich ascites cells in bicarbonate buffered media*

Aerobic glycolysis and oxygen uptake were determined simultaneously by the method of Dixon and Keilin (1933) using Barcroft differential manometers. The precautions described by Elliott and Schroeder (1934) and Dixon (1951) were observed. Measurements were made at 37° C. and the flasks were gassed with water-saturated 95 per cent $O_2$ + 5 per cent $CO_2$ during a 15 minute equilibration period. The side arms contained 0·5 ml. of 1·0 M HCl and the stopper cavities were filled with 40 per cent KOH. Clerici solution ($P_0 = 2500$) was used in the manometers.

The two flasks of each manometer contained identical aliquots of either (*a*) the Ehrlich ascites cell suspension in medium incubated with tissue but not irradiated, or (*b*) the Ehrlich ascites cell suspension in medium incubated with mouse abdominal wall tissue after 1000 roentgen X-irradiation.

Experiments were conducted over periods of 40–50 minutes during which time the rates of glycolysis and respiration remained apparently undiminished.

The pH at the beginning and end of each experiment was calculated using the Henderson-Hassalbalch equation with the values $pK^1 = 6{\cdot}1$, $\alpha = 0{\cdot}51$ (c.c. $CO_2$ dissolved per ml. of medium at 37° C. and 760 mm.) for ascites serum and $pK^1 = 6{\cdot}32$, $\alpha = 0{\cdot}57$ for non-proteinaceous media. The initial pH values of all suspensions were found to lie within the range pH 7·2–7·4.

Retention curves necessary to correct the observed glycolysis for the amount of $CO_2$ retained by the medium and not registered on the manometer were determined by the method of Brekke and Dixon (1937). The values of $R/\Delta$ pH for the X-irradiated medium were on an average 14 per cent lower than for the corresponding non-irradiated medium.

*Oxygen uptake and P/O ratio of Ehrlich ascites cell mitochondria in phosphate buffered media incubated with mouse antero-lateral abdominal wall*

Corresponding series of Warburg flasks were set up with (a) 2·0 ml. aliquots of phosphate buffered medium incubated with mouse tissue but not irradiated, and (*b*) 2·0 ml. aliquots of corresponding medium incubated with tissue after 1000 roentgen X-irradiation. To each flask was added 0·26 ml. of the following solution : KF, 0·173 M ; $MgCl_2$, 0·023 M ; ADP, 0·029 M ; AMP, 0·012 M ; EDTA, 0·023 M ; cytochrome-c, $1{\cdot}13 \times 10^{-4}$ M ; DPN, 0·0028 M. The side arms of each flask contained 0·24 ml. of 0·3 M glucose plus 1–2 mg. of crystalline hexokinase, and the centre wells 0·2 ml. of 40 per cent KOH solution with a roll of filter paper.

After cooling the flasks in ice, 0·4 ml. of the mitochondrial suspension in 0·25 M. sucrose was added to each vessel.

Final concentrations in each flask were as follows: potassium phosphate buffer (pH 7·4), 0·045 M; KF, 0·015 M; $MgCl_2$, 0·008 M; ADP, 0·0025 M; AMP, 0·001 M; EDTA, 0·002 M; glucose, 0·034 M; KCl, 0·01 M; sucrose 0·062 M; DPN, $2{\cdot}4 \times 10^{-4}$ M; cytochrome-c, $9{\cdot}8 \times 10^{-6}$ M; hexokinase, 1–2 mg. (plus unknown amounts of lactic acid, amino acids, substrates, etc., resulting from incubation of part of the flask contents with mouse antero-lateral abdominal wall).

Experiments were conducted at 30° C. over periods of 25–35 minutes with air as the gas phase. An equilibration time of 13 minutes on the manometer bath was allowed before closing the taps and adding the side-arm contents. The reaction was stopped by addition of 1·0 ml. of 30 per cent TCA to each flask which was then placed in ice. Phosphate uptake was determined by the method of Fiske and Subbarow (1925) on the cold TCA filtrate.

*Amino acid analysis*

Samples of corresponding non-irradiated and X-irradiated media were deproteinised by addition of 4 vols. ethanol. After standing overnight at 4° C. and centrifuging, 0·2 ml. aliquots of the supernatant were analysed for total free amino acid and peptide content by the quantitative ninhydrin method of Troll and Cannan (1953) using a standard leucine curve.

*Protein analysis*

Duplicate aliquots of corresponding non-irradiated and X-irradiated media were analysed for total protein content according to the biuret method of Gornall, Bardawill and David (1949).

*Assay of materials*

*Hexokinase* was used in the crystalline form supplied by General Biochemicals Inc., Chagrin Falls, Ohio, and was assayed (*a*) by hexose disappearance according to Somogyi (1952) and (*b*) by measurement of acid labile phosphorus disappearance according to Crane and Sols (1955). Preparations were found to have an activity of approximately 150 units per mg.

*Cytochrome-c* was used as a 1 per cent solution in isotonic saline as supplied by General Biochemicals Inc. and was found to contain $5{\cdot}87 \times 10^{-7}$ moles per ml. when assayed according to Umbreit, Burris and Stauffer (1957).

*AMP* and *ADP* were used as supplied by General Biochemicals Inc. and by the H.M. Chemical Co. of Los Angeles, California, respectively.

*DPN* was obtained from General Biochemicals Inc. and was assayed 85 per cent pure by a modification of the dithionite reduction method of Brodie (1955) using 0·2 M phosphate buffer instead of 0·1 M.

All other substrates and chemicals were of Analytical Reagent grade, and where necessary were neutralised to pH 7·4 with potassium hydroxide before use.

### RESULTS

### (1) *Aerobic glycolysis of Ehrlich ascites cells in ascites plasma incubated with mouse antero-lateral abdominal wall*

The aerobic glycolysis of Ehrlich ascites cells was compared in 6 corresponding batches of ascites plasma incubated with abdominal wall tissue from healthy mice (*a*) non-irradiated and (*b*) 1000 roentgen X-irradiated before incubation.

The results in Table I show that the glycolysis was on an average 15 per cent lower in the X-irradiated medium.

TABLE I.—*Aerobic Glycolysis of Ehrlich Ascites Cells in Ascites Plasma Incubated with Tissue,* (a) *Not Irradiated and* (b) 1000 *Roentgen X-Irradiated before Incubation*

| Batch No. | Glycolysis $Q_G^{O_2}$ Not irradiated | X-irradiated | Percentage difference |
|---|---|---|---|
| 1 | 39·5 | 36·6 | −7·3 |
| | 30·2 | 31·6 | +4·6 |
| 2 | 36·6 | 27·9 | −23·8 |
| | 41·4 | 20·2 | −51·2 |
| 3 | 23·0 | 21·5 | −5·8 |
| | 24·8 | 22·2 | −10·5 |
| 4 | 31·1 | 24·8 | −12·7 |
| | 28·6 | 24·3 | −15·0 |
| 5 | 31·8 | 30·3 | −4·7 |
| | 38·6 | 27·5 | −28·7 |
| 6 | 31·8 | 26·1 | −17·9 |
| | 31·0 | 27·6 | −11·0 |
| Average | 32·4±4·4* | 26·7±3·5 | −(15·3±10·1) |

* Average deviation from mean.

(2) *Oxygen uptake of Ehrlich ascites cells in ascites plasma incubated with mouse antero-lateral abdominal wall*

The oxygen uptake of Ehrlich ascites cells was compared in 5 corresponding batches of ascites plasma incubated with abdominal wall tissue from healthy mice (*a*) non-irradiated and (*b*) 1000 roentgen X-irradiated before incubation.

The results in Table II show that the oxygen uptake was on an average 43 per cent higher in the media X-irradiated with tissue before incubation.

TABLE II.—*Oxygen Uptake of Ehrlich Ascites Cells in Ascites Plasma Incubated with Tissue* (a) *Not Irradiated and* (b) 1000 *Roentogen X-irradiated before Incubation*

| Batch No. | Oxygen uptake $Q_{O_2}$ Not irradiated | X-irradiated | Percentage difference |
|---|---|---|---|
| 1 | 2·01 | 4·55 | +126·3 |
| | 3·03 | 6·04 | +99·3 |
| 2 | 6·57 | 7·26 | +10·5 |
| | 4·78 | 5·77 | +20·7 |
| 3 | 6·07 | 8·10 | +33·4 |
| | 5·38 | 7·53 | +40·0 |
| 4 | 5·54 | 6·68 | +20.6 |
| | 6·70 | 8·92 | +33·1 |
| 5 | 5·84 | 7·32 | +25·4 |
| | 6·25 | 7·63 | +22·1 |
| Average | 5·22±1·17* | 6·98±0·98 | +(43·1±27·8) |

* Average deviation from mean.

(3) *Aerobic glycolysis of Ehrlich ascites cells in ascites plasma incubated with antero-lateral abdominal wall from tumour-bearing mice*

For these experiments the antero-lateral abdominal wall was removed from mice bearing a 10-day Ehrlich ascites tumour. Any small areas of solid tumour formation were carefully removed and macroscopically the tissue appeared normal. Incubation with ascites plasma was carried out as described for antero-lateral abdominal wall from healthy mice.

The aerobic glycolysis of Ehrlich ascites cells was then compared in 3 batches of ascites plasma incubated with the tissue (*a*) non-irradiated and (*b*) 1000 roentgen X-irradiated before incubation.

The results in Table III show that the glycolysis was on an average very slightly lower in the X-irradiated medium than in the corresponding non-irradiated control.

TABLE III.—*Aerobic Glycolysis of Ehrlich Ascites Cells in Ascites Plasma Incubated with Antero-lateral Abdominal Wall from Tumour-bearing Mice* (a) *Not Irradiated and* (b) 1000 *Roentgen X-irradiated before Incubation*

| Batch No. | Glycolysis $Q_G^{O_2}$ Not irradiated | X-irradiated | Percentage difference |
|---|---|---|---|
| 1 | 33·2 | 29·2 | −12·0 |
| | 38·1 | 37·1 | −2·6 |
| 2 | 32·8 | 33·5 | +2·1 |
| 3 | 35·2 | 33·8 | −4·0 |
| | 35·0 | 35·2 | +0·6 |
| Average | 34·9±1·5* | 33·8±1·9 | −(3·3±2·8) |

* Average deviation from mean.

(4) *Oxygen uptake of Ehrlich ascites cells in ascites plasma incubated with antero-lateral abdominal wall from tumour-bearing mice*

Antero-lateral abdominal wall was removed from mice bearing a 10-day Ehrlich ascites tumour, and was incubated with ascites plasma as above.

The oxygen uptake of Ehrlich ascites cells was then compared in 2 batches of ascites plasma incubated with the tissue (*a*) non-irradiated and (*b*) 1000 roentgen X-irradiated before incubation.

The results in Table IV show that the oxygen uptake was on an average 50 per cent higher in the medium incubated with tissue after X-irradiation. The magnitude of the effect decreased with storage time of the media before use, indicating lability of the factor(s) responsible.

(5) *Oxygen uptake of Ehrlich ascites cells in ascites plasma deproteinised after incubation with mouse antero-lateral abdominal wall*

Ehrlich ascites plasma fortified with potassium succinate was incubated with healthy mouse antero-lateral abdominal wall (*a*) without radiation and (*b*) with 1000 roentgen X-irradiation before incubation. The media were subsequently deproteinised.

The oxygen uptake of Ehrlich ascites cells was then compared in 4 corresponding batches of medium prepared with and without radiation.

TABLE IV.—*Oxygen Uptake of Ehrlich Ascites Cells in Ascites Plasma Incubated with Antero-lateral Abdominal Wall from Tumour-bearing Mice* (a) *Not Irradiated and* (b) 1000 *Roentgen X-irradiated Before Incubation*

| Batch No. | Oxygen uptake $Q_{O_2}$ Not irradiated | X-irradiated | Percentage difference | Storage time† after incubation (days) |
|---|---|---|---|---|
| 1 | 3·96 | 5·58 | +41·0 | 5 |
| 2 | 3·00 | 7·80 | +160·0 | 3 |
| | 4·72 | 4·91 | +4·0 | 8 |
| | 5·39 | 6·21 | +17·0 | 10 |
| Average | 4·27±0·79* | 6·13±0·88 | +(50·5±9·5) | — |

* Average deviation from mean.
† Media were stored at −30° C. before experiment.

The results in Table V show that the oxygen uptake was on an average 32 per cent higher in the medium incubated with tissue after X-irradiation.

TABLE V.—*Oxygen uptake of Ehrlich Ascites Cells in Ascites Plasma Deproteinised After Incubation with Mouse Antero-lateral Abdominal Wall* (a) *Not Irradiated and* (b) 1000 *Roentgen X-irradiated before Incubation*

| Batch No. | Oxygen uptake $Q_{O_2}$ Not irradiated | X-irradiated | Percentage difference |
|---|---|---|---|
| 1 | 9·00 | 10·93 | +21·5 |
| 2 | 4·15 | 5·78 | +39·2 |
| | 5·15 | 6·85 | +33·0 |
| 3 | 7·05 | 11·10 | +57·5 |
| | 7·65 | 9·90 | +29·6 |
| 4 | 9·83 | 11·82 | +20·4 |
| | 8·86 | 10·64 | +20·2 |
| Average | 7·38±1·66* | 9·57±1·86 | +(31·6±9·9) |

* Average deviation from mean.

(6) *Oxygen uptake of Ehrlich ascites cells in Ringer bicarbonate solution incubated with mouse antero-lateral abdominal wall*

In these experiments Ringer bicarbonate fortified with potassium succinate (solution (*b*)) was incubated with healthy mouse antero-lateral abdominal wall (*a*) without radiation and (*b*) with 1000 roentgen X-irradiation before incubation.

The oxygen uptake of Ehrlich ascites cells was then compared in 4 corresponding batches of medium prepared with and without radiation.

The results in Table VI show that the oxygen uptake was on an average 14 per cent higher in the medium incubated with tissue after X-irradiation.

(7) *Oxygen uptake of Ehrlich ascites cells in ascites plasma deproteinised after* 3 *hours' incubation at* 37° *C. without tissue*

Batches of succinate fortified Ehrlich ascites plasma were divided into two portions, one of which was given 1000 roentgen X-irradiation. Both samples were

TABLE VI.—*Oxygen Uptake of Ehrlich Ascites Cells in Ringer Bicarbonate Solution Incubated with Mouse Antero-lateral Abdominal Wall* (a) *Not Irradiated and* (b) 1000 *Roentgen X-irradiated before Incubation*

| Batch No. | Oxygen uptake $Q_{O_2}$ Not irradiated | X-irradiated | Percentage difference |
|---|---|---|---|
| 1 | 11·89 | 12·41 | +4·4 |
| 2 | 6·92 | 8·72 | +26·1 |
| 3 | 9·33 | 10·62 | +13·8 |
| | 9·16 | 10·02 | +9·4 |
| 4 | 6·96 | 7·90 | +13·5 |
| | 7·02 | 8·34 | +18·8 |
| Average | 8·55±1·58* | 9·67±1·35 | +(14·3±5·4) |

* Average deviation from mean.

incubated 3 hours at 37° C. under $O_2/CO_2$ gas mixture and subsequently deproteinised.

The oxygen uptake of Ehrlich ascites cells was compared in 4 corresponding batches of the deproteinised fluid prepared with and without radiation.

The results in Table VII show that the oxygen uptake was almost identical in corresponding non-irradiated and X-irradiated media.

TABLE VII.—*Oxygen Uptake of Ehrlich Ascites Cells in Ascites Plasma Deproteinised after* 3 *hours' Incubation at* 37° *C. Without Tissue* (a) *Not Irradiated and* (b) 1000 *Roentgen X-irradiated before Incubation*

| Batch No. | Oxygen uptake $Q_{O_2}$ Not irradiated | X-irradiated | Percentage difference |
|---|---|---|---|
| 1 | 9·10 | 9·72 | +6·8 |
| 2 | 8·65 | 8·72 | +0·8 |
| | 11·20 | 11·05 | −1·3 |
| 3 | 9·78 | 9·64 | −1·4 |
| | 8·95 | 8·50 | −5·0 |
| 4 | 9·30 | 9·10 | −2·1 |
| | 7·10 | 7·05 | −0·7 |
| Average | 9·15±0·80* | 9·11±0·88 | −(0·4±2·2) |

* Average deviation from mean.

(8) *Oxygen uptake of Ehrlich ascites cells in phosphate buffered media incubated with mouse antero-lateral abdominal wall*

Three distinct series of experiments were carried out using phosphate buffered media.

(1) Phosphate buffered medium containing excess glucose (solution (*d*)) was incubated with healthy mouse antero-lateral abdominal wall (*a*) without radiation and (*b*) with 1000 roentgen X-irradiation before incubation. The oxygen uptake of Ehrlich ascites cells was then compared in the media prepared with and without radiation.

(2) As above, except that 0·5 ml. of 0·3 M potassium succinate was added to each 70 ml. of phosphate buffered medium (*d*) either before or after incubation with mouse tissue.

(3) As above, except that Ringer phosphate-succinate medium (*c*), i.e. containing no glucose, was used for incubation witn the mouse tissue.

Oxygen uptake of ascites cells was measured in triplicate in each batch of corresponding non-irradiated and X-irradiated media. Experiments were conducted at 37° C. over periods of 40 minutes, during which time respiration remained undiminished. The gas phase was pure oxygen.

Table VIII shows that in phosphate buffer with glucose as substrate, the oxygen uptake of ascites cells was on an average 14·7 per cent lower in the medium incubated with tissue after X-irradiation. In phosphate buffer containing glucose and succinate as substrates, the effect was less pronounced with 4·4 per cent average lowering of oxygen uptake in the X-irradiated medium. Only 1 per cent average lowering was observed in phosphate medium with no glucose but succinate added prior to incubation.

TABLE VIII.—*Oxygen Uptake of Ehrlich Ascites Cells in Phosphate Buffered Media Incubated with Mouse Antero-lateral Abdominal Wall* (a) *Not Irradiated and* (b) 1000 *Roentgen X-irradiated before Incubation*

| Medium and substrate | No. of corresponding batches | No. of experiments | Oxygen uptake $Q_{O_2}$ Not irradiated | Oxygen uptake $Q_{O_2}$ X-irradiated | Percentage difference |
|---|---|---|---|---|---|
| Phosphate buffered medium (*d*) (glucose) | 2 | 6 | 4·71±0·31 | 4·02±0·23* | −(14·7±0·8) |
| Phosphate buffered medium (*d*) (glucose + succinate) | 3 | 9 | 5·62±0·31 | 5·38±0·27 | −(4·4±0·3) |
| Ringer-phosphate medium (*c*) (succinate) | 3 | 9 | 10·08±0·42 | 9·99±0·40 | −(1·0±0·8) |

* Average deviation from mean.

(9) *Oxygen uptake of Ehrlich ascites cells in Ringer phosphate solution incubated for various times with mouse antero-lateral abdominal wall*

The $Q_{O_2}$ of Ehrlich ascites cells was measured in succinate-fortified Ringer phosphate solution (*c*) which had been incubated with healthy mouse antero-lateral abdominal wall for varying times.

The results in Table IX show that, within the limits of experimental accuracy, the $Q_{O_2}$ values of ascites cells were identical in media incubated between 1–5 hours with mouse tissue.

(10) *Oxygen uptake of Ehrlich ascites cell mitochondria in phosphate buffered medium incubated with mouse antero-lateral abdominal wall*

The oxygen uptake of Ehrlich ascites cell mitochondria was compared in 7 corresponding batches of phosphate buffered medium incubated with healthy mouse tissue (*a*) without radiation and (*b*) with 1000 roentgen X-irradiation before incubation. Media were triturated to pH 7·5 with KOH after incubation.

Mitochondria showed a negligible endogenous oxygen uptake in phosphate buffered medium which had not been incubated with mouse antero-lateral abdominal wall and to which no citric acid cycle substrates had been added. However

TABLE IX.—*Oxygen Uptake of Ehrlich Ascites Cells in Ringer Phosphate Solution Incubated for* 1–8 *hours with Mouse Antero-lateral Abdominal Wall. No Irradiation*

| Time of incubation (hours) | Oxygen uptake $Q_{O_2}$ | Average $Q_{O_2}$ | Percentage increase above control |
|---|---|---|---|
| 0 (Control) | 7·1<br>7·4 | 7·3 | — |
| 1 | 7·7<br>8·1 | 7·9 | 8.2 |
| 2 | 7·9 | 7·9 | 8·2 |
| 3 | 7.6<br>8·2 | 7.9 | 8.2 |
| 5 | 7·9<br>7·8 | 7·9 | 8·2 |
| 8 | 8·2<br>8·8 | 8·5 | 16·5 |

the mitochondria showed considerable oxygen uptake in the same medium after incubation with mouse tissue. This was apparently due to liberation of oxidisable substrates, from the tissue into the medium during incubation.

The results in Table X show that the oxygen uptake of Ehrlich ascites mitochondria was on an average 20 per cent lower in the medium incubated with tissue after irradiation, than in the control incubation medium prepared without irradiation.

As an additional part of each experiment the biochemical intactness of the mitochondria was checked by measuring their oxygen uptake in phosphate buffered medium fortified with 0·01 M potassium succinate as substrate (cf. Hawtrey and Silk, 1960). Batches showing $Q_{O_2}$ values in the range 85–130 were used for experiment.

TABLE X.—*Oxygen Uptake of Ehrlich Ascites Cell Mitochondria in Phosphate Buffered Medium Incubated with Mouse Antero-lateral Abdominal Wall* (a) *Not Irradiated and* (b) 1000 *Roentgen X-irradiated before Incubation*

| Batch No. | Oxygen uptake $Q_{O_2}$ | | Percentage difference |
|---|---|---|---|
| | Not irradiated | X-irradiated | |
| 1 | 62·7<br>— | 52·8<br>56·4 | −12·9 |
| 2 | 100·1<br>93·0 | 81·0<br>79·3 | −17·0 |
| 3 | 38·7<br>38·6 | 30·8<br>32·8 | −17·7 |
| 4 | 41·9<br>47·5 | 34·1<br>32·7 | −25·3 |
| 5 | 70·5<br>96·2 | 56·8<br>58·4 | −30·9 |
| 6 | 94·9<br>77·6 | 80·5<br>71·7 | −11·0 |
| 7 | 76·1<br>72·0 | 58·2<br>47·0 | −29·0 |
| Average | 70·0±18·5* | 55·2±14·4 | −(20·5±6·7) |

* Average deviation from mean.

(11) *P/O ratio of Ehrlich Ascites cell mitochondria in phosphate buffered medium incubated with mouse antero-lateral abdominal wall*

As part of the experiments to measure $O_2$ uptake of Ehrlich ascites cell mitochondria, the *P/O* ratios of these particulates were determined in 7 corresponding batches of phosphate buffered medium incubated with healthy mouse tissue (*a*) without radiation and (*b*) with 1000 roentgen X-irradiation before incubation.

The results in Table XI show that the *P/O* ratio was on an average 16 per cent lower in the medium incubated with tissue after X-irradiation. Since no citric acid cycle metabolites were added at any stage in the experiments, the *P/O* ratios shown are those of the substrate(s) liberated from the mouse tissue into the phosphate buffered medium during incubation.

TABLE XI.—*P/O Ratio of Ehrlich Ascites Cell Mitochondria in Phosphate Buffered Medium Incubated with Mouse Antero-lateral Abdominal Wall* (a) *Not Irradiated and* (b) 1000 *Roentgen X-irradiated before Incubation*

| Batch No. | *P/O* ratio: Not irradiated | *P/O* ratio: X-irradiated | Percentage difference |
|---|---|---|---|
| 1 | 1·16<br>— | 1·11<br>0·97 | −10·4 |
| 2 | 1·13<br>1·14 | 1·09<br>1·01 | −8·1 |
| 3 | 1·14<br>1·11 | 1·10<br>0·97 | −8·0 |
| 4 | 1·24<br>1·08 | 0·84<br>0·87 | −26·7 |
| 5 | 1·29<br>1·15 | 0·94<br>1·01 | −20·5 |
| 6 | 0·91<br>— | 0·84<br>0·77 | −12·0 |
| 7 | 0·98<br>1·14 | 0·85<br>0·71 | −26·4 |
| Average | 1·12±0·07* | 0·93±0·10 | −(16·0±7·3) |

* Average deviation from mean.

(12) *Effect of radiation on the protein and amino acid analysis of media after incubation with mouse antero-lateral abdominal wall*

Table XII shows the total protein content, and the content of free amino acids and peptides present in corresponding batches of various media incubated with mouse tissue. In all cases the incubation medium prepared after X-irradiation showed a lower protein content and a higher free amino acid and peptide content than the corresponding non-irradiated medium.

### DISCUSSION

Previous work (Silk, Hawtrey and Macintosh, 1958) showed that irradiation of the normal tumour-bed and stroma cells of a neoplasm, might "indirectly" affect the respiratory metabolism of non-irradiated malignant cells. The "indirect" effect was considered due to radiation induced substances from the normal cells reaching the malignant cells via the common environment of peripheral lymph.

TABLE XII.—*Protein and Amino Acid Analysis of Media after Incubation with Mouse Antero-lateral Abdominal Wall* (a) *without Irradiation and* (b) *with* 1000 *Rœntgen X-irradiation Prior to Incubation*

| Medium | No. of corres-ponding batches | Total protein (mg./ml.) Not irradiated | Total protein (mg./ml.) X-irradiated | Total protein (mg./ml.) Percentage difference | Total free amino acids and peptides (mg./ml.) Not irradiated | Total free amino acids and peptides (mg./ml.) X-irradiated | Total free amino acids and peptides (mg./ml.) Percentage difference |
|---|---|---|---|---|---|---|---|
| Ascites plasma incubated with mouse tissue | 5 | $8 \cdot 85 \pm 0 \cdot 57$* | $7 \cdot 83 \pm 1 \cdot 23$ | $-(15 \cdot 8 \pm 12 \cdot 6)$ | $1 \cdot 25 \pm 0 \cdot 21$ | $1 \cdot 30 \pm 0 \cdot 14$ | $+(6 \cdot 0 \pm 15 \cdot 8)$ |
| Ascites plasma deproteinised after incubation with mouse tissue | 4 | — | — | — | $0 \cdot 88 \pm 0 \cdot 37$ | $0 \cdot 96 \pm 0 \cdot 14$ | $+(48 \cdot 7 \pm 60 \cdot 4)$ |
| Ascites plasma deproteinised after incubation without tissue | 3 | — | — | — | $0 \cdot 08 \pm 0 \cdot 16$ | $1 \cdot 04 \pm 0 \cdot 17$ | $+(26 \cdot 0 \pm 7 \cdot 7)$ |
| Phosphate buffered medium (*d*) incubated with mouse tissue | 8 | $1 \cdot 02 \pm 0 \cdot 18$ | $0 \cdot 85 \pm 0 \cdot 21$ | $-(16 \cdot 6 \pm 7 \cdot 2)$ | $0 \cdot 45 \pm 0 \cdot 13$ | $0 \cdot 58 \pm 0 \cdot 17$ | $+(28 \cdot 6 \pm 6 \cdot 7)$ |

* Average deviation from mean.

For the present investigation, mouse antero-lateral abdominal wall tissue has been selected to represent part of the normal tumour-bed and stroma of the Enrlich ascites tumour. Various physiological media have been incubated with this tissue to provide the environmental fluids through which X-irradiation has been found to influence the respiratory metabolism of non-irradiated ascites cells and mitochondria.

In bicarbonate buffered media, intact ascites cells showed slightly lower aerobic glycolysis (Tables I and III) but markedly higher oxygen uptake (Tables II, IV, V and VI) in the X-irradiated medium than in the corresponding non-irradiated control. No elevation of cell respiration was observed in the case of ascites plasma deproteinised after X-irradiation and incubation in the absence of mouse tissue (Table VII).

In phosphate buffered media, the oxygen uptake of ascites cells was lower in the medium prepared with X-irradiation prior to incubation (Table VIII). The effect is therefore opposite to that found in bicarbonate buffered media. A similar inversion of effect was observed in previous work (Silk, Hawtrey and Macintosh, 1958) and may be due to competition between respiratory and glycolytic processes for the low amount of inorganic phosphate usually available in physiological bicarbonate media (cf. Kvamme, 1958).

The respiration and *P*/*O* ratio of ascites cells mitochondria were also found to be depressed in the phosphate buffered medium incubated with mouse tissue after X-irradiation (Tables X and XI). The effect therefore parallels that observed for intact cells in phosphate media, but is again opposite to that observed for whole cells in bicarbonate buffered media.

Due to protein radiolysis, the X-irradiated incubation medium has, in each case, a lower protein content but a higher free amino acid and peptide content than the corresponding non-irradiated medium (Table XII). This holds for X-irradiated and non-irradiated ascites plasma incubated in the absence of mouse tissue, yet the oxygen uptake of ascites cells was not different in the two media (Table VII). Thus radiolysis of the environmental medium itself would not account for the observed effects on respiratory metabolism.

The media incubated with mouse abdominal wall contain respiratory substrates liberated by the tissue. This is evidenced by the high $Q_{O_2}$ of mitochondria in phosphate buffered media incubated without added substrates such as succinate (Table X) and by the fact that ascites cells show a higher $Q_{O_2}$ in Ringer phosphate-succinate solution incubated with mouse tissue, than in the same medium without incubation (Table IX). The amount of substrates liberated from the tissue during incubation times of 1–5 hours was adequate to maintain the respiration rate of ascites cells at a consistent level above the control value (Table IX).

A mere difference in quantity of liberated substrates would not however account for the difference in rates of oxygen uptake shown by ascites cells and mitochondria in corresponding batches of non-irradiated and X-irradiated media. It would therefore seem that the X-irradiated medium contains in addition some radiation induced factor(s) capable of affecting the rate of oxygen consumption. Table VIII shows that liberation of these factors may depend upon the presence of glucose in the medium incubated with mouse tissue.

Bernheim, Barber, Ottolenghi and Wilbur (1958) found that certain tissues with no natural anti-oxidants form peroxides on aerobic incubation, whereas other tissues do this if the natural anti-oxidant is destroyed by radiation. Shuster

(1955) showed that methyl linoleate and linolenate hydroperoxides inhibit the oxygen uptake of Ehrlich ascites cells in Ringer phosphate solution and inhibit their glycolysis in Ringer bicarbonate solution. Such perioxides also inhibit the oxidising enzymes in liver mitochondria (Ottolenghi, Bernheim and Wilbur, 1955).

Our findings concerning the radiation effect on glycolysis in bicarbonate media (Tables I and III) and on oxygen uptake in phosphate media (Tables VIII and X) are therefore compatible with the presence of peroxides in the X-irradiated medium. The elevation of oxygen uptake observed in bicarbonate buffered X-irradiated medium (Tables II, IV, V and VI) does not however appear to be compatible with the known effects of peroxides on respiration. The X-irradiated media were examined for organic hydroperoxides using both iodide and titanous sulphate reagents (Scholes, Weiss and Wheeler, 1956) but none were found present. Spectroscopic examination of corresponding non-irradiated and X-irradiated media revealed no qualitative difference in pattern in the region 200–1800 m$\mu$.

It may thus be concluded that the environmental fluid incubated with an irradiated normal tissue representing the tumour-bed of a neoplasm, contains substances which affect the respiratory metabolism of non-irradiated malignant cells. The factors responsible for this " indirect " effect are at present unknown, but do not result from radiolysis of the environmental fluid itself and must therefore originate from metabolism of the tissue irradiated in contact with this fluid.

### SUMMARY

1. Various proteinaceous and non-proteinaceous physiological media were incubated 3 hours at 37° C with mouse antero-lateral abdominal wall tissue (*a*) without radiation and (*b*) with 1000 roentgen X-radiation of the medium plus tissue prior to incubation.

2. The repiratory and glycolytic metabolism of Ehrlich ascites cells and mitochondria was then compared in corresponding batches of media prepared with and without radiation. In some experiments oxidisable substrates were added to the media, while in others substrates liberated from the mouse abdominal wall tissue served to maintain respiration of the added ascites cells or mitochondria.

3. With bicarbonate buffering, intact ascites cells showed lower aerobic glycolysis and markedly elevated oxygen uptake in the X-irradiated medium.

4. With phosphate buffering the opposite effect was observed. Both intact ascites cells and mitochondria showed lower oxygen uptake in the X-irradiated medium. Mitochondria showed lower $P/O$ ratios in the X-irradiated medium than in the corresponding non-irradiated control.

5. Since neither the cells nor the mitochondria were directly radiated, the results demonstrate an " indirect " effect of X-radiation on respiratory metabolism. The effect operates via the environmental medium irradiated in contact with a tissue selected to represent the normal tumour-bed and stroma of the Ehrlich tumour.

6. The " indirect " effect does not seem due to radiolysis of the environmental medium itself, nor do organic hydroperoxides appear to be responsible. It is suggested that metabolic products from the irradiated tissue are liberated into the medium, and that these influence the respiration rate of added Ehrlich ascites cells and mitochondria.

This work has formed part of the research being undertaken in terms of a Senior Fellowship awarded to one of us (M. H. S.) by the National Cancer Association of South Africa.

### REFERENCES

BERNHEIM, F., BARBER, A. A., OTTOLENGHI, A. AND WILBUR, K. M.—(1958) *Radiation Res.* **9**, 91.
BREKKE, B. AND DIXON, M.—(1937) *Biochem J.*, **31**, 2000.
BRODIE, A. F.—(1955) *Methods in Enzymology*, **2**, 693.
CLELAND, K. W. AND SLATER, E. C.—(1953) *Biochem. J.*, **53**, 548.
CRANE, R. K. AND SOLS, A.—(1955) *Methods in Enzymology*, **1**, 277.
DIXON, M.—(1951) ' Manometric Methods '. Cambridge University Press, 3rd Ed., p. 105.
*Idem* AND KEILIN, D.—(1933) *Biochem. J.* **27**, 86.
ELLIOTT, K. A. C. AND SCHROEDER, E. F.—(1934) *Ibid.*, **28**, 1920.
FISKE, C. H. AND SUBBAROW, Y.—(1925) *J. biol. Chem.*, **66**, 375.
GORNALL, A. G., BARDAWILL, C. J. AND DAVID, M. M.—(1949) *Ibid.*, **177**, 751.
HAWTREY, A. O. AND SILK, M. H.—(1960) *Biochem. J.*, **74**, 21.
KVAMME, E.—(1958) *Acta physiol. scand.*, **42**, 204.
OTTOLENGHI, A., BERNHEIM, F. AND WILBUR, K. M.—(1955) *Arch. Biochem. Biophys.*, **56**, 157.
SCHOLES, G., WEISS, J. AND WHEELER, C. M.—(1956) *Nature, Lond.*, **178**, 157.
SHUSTER, C. W.—(1955) *Proc. Soc. exp. Biol., N.Y.*, **90**, 423.
SILK, M. H., HAWTREY, A. O. AND MACINTOSH, I. J. C.—(1958) *Cancer Res.*, **18**, 1257.
SOMOGYI, M.—(1952) *J. biol. Chem.*, **195**, 19.
TROLL, W. AND CANNAN, R. K.—(1953) *Ibid.*, **200**, 803.
UMBREIT, W. W., BURRIS, R. H. AND STAUFFER, J. F.—(1957) ' Manometric Techniques '. Minnesota (Burgess Pub. Co.), 3rd Ed., p. 300.